# Changes in Age Distribution of Obesity-Associated Cancers

**DOI:** 10.1001/jamanetworkopen.2019.9261

**Published:** 2019-08-14

**Authors:** Siran M. Koroukian, Weichuan Dong, Nathan A. Berger

**Affiliations:** 1 Department of Population and Quantitative Health Sciences, Case Western Reserve University School of Medicine, Cleveland, Ohio; 2 Case Comprehensive Cancer Center, Case Western Reserve University School of Medicine, Cleveland, Ohio

## Abstract

**Importance:**

Recent studies have documented an association between overweight and obesity and certain cancers, as well as an increased incidence of obesity-associated cancers (OACs) in younger individuals. However, although important for cancer-control efforts, it is not known which subgroups of the population are most affected by these changes.

**Objective:**

To examine temporal shifts in age distribution of OACs and non-OACs across race/ethnicity- and sex-specific strata.

**Design, Setting, and Participants:**

This population-based, cross-sectional study assessed individuals residing in Surveillance, Epidemiology, and End Results sites who were diagnosed with incident OACs and non-OACs from January 1, 2000, to December 31, 2016. Data analysis was performed from August 1, 2018, to June 30, 2019.

**Exposure:**

Study years.

**Main Outcomes and Measures:**

Changes in the age distribution of incident cases across race/ethnicity- and sex-specific strata over time. For all OACs and non-OACs, changes in the incidence rates, the number of incident cases, and the distribution of the cases across population subgroups were studied. Race/ethnicity- and sex-stratified logistic regression analysis was performed to determine whether the annual change in the odds associated with an age group increased (or decreased) to a greater (or lesser) extent in OACs than in non-OACs. Heat maps were created to highlight the change in the number of cases during the study period for each OAC and select non-OACs.

**Results:**

The study population included 2 665 574 incident OAC cases (70.3% women) and 3 448 126 incident non-OAC cases (32.0% women). From 2000 to 2016, the distribution by age showed that the percentage of incident cases increased in the 50- to 64-year age group for both OACs and non-OACs. The increase in the number of OACs in this age group ranged from 25.3% in non-Hispanic white women to 197.8% in Hispanic men. The change in the number of OACs in the 20- to 49-year age group ranged from −5.9% in non-Hispanic white women to 94.6% in Hispanic women, and the increase in the number of OACs in the 65 years or older group ranged from 2.5% in non-Hispanic white women to 102.0% in Hispanic women. Logistic regression analysis revealed a greater annual increase in the odds for OACs than for non-OACs for individuals in the 50- to 64-year age group but a decrease for individuals in the 65 years or older group. Among men in the 50- to 64-year age group, the OAC to non-OAC ratio of odds ratios (ORs) ranged from 1.005 (95% CI, 1.002-1.008) in non-Hispanic black men to 1.013 (95% CI, 1.012-1.014) in non-Hispanic white men, implying that the annual increase was 0.5% higher for OACs than for non-OACs in non-Hispanic black men and 1.3% higher for OACs than for non-OACs in non-Hispanic white men. Among women in the 50- to 64-year age group, the OAC to non-OAC ratio of ORs ranged from 1.002 (95% CI, 0.999-1.006) in Hispanic women to 1.005 (95% CI, 1.002-1.009) in non-Hispanic black women. In men and women aged 65 or older, the OAC vs non-OAC ratio of ORs was consistently less than 1.000 for all race/ethnicity groups, indicating that whereas the OAC group experienced a decrease in this age group, the non-OACs experienced either a smaller decrease or an increase over time.

**Conclusions and Relevance:**

This study indicates that from 2000 to 2016, a shift toward younger age groups occurred in incident OACs. The findings have important public health implications and suggest that interventions to reduce obesity and to implement individualized screening programs are needed.

## Key Points

**Question:**

What are the temporal shifts in age distribution for obesity-associated cancers across sex- and race/ethnicity-specific strata?

**Findings:**

In this cross-sectional study of 2 665 574 incident obesity-associated cancer cases and 3 448 126 incident non–obesity-associated cancer cases from 2000 to 2016, the percentage of individuals diagnosed with incident obesity-associated cancers increased in younger age groups, with some of the greatest increases observed for liver and thyroid cancers (all sex- and race/ethnicity-specific strata), gallbladder and other biliary cancers (non-Hispanic black men and women and Hispanic men), and uterine cancer (in Hispanic women in the 50- to 64-year age group).

**Meaning:**

The findings suggest that there has been a shift of obesity-associated cancer burden to younger age groups and that interventions to reduce obesity and to implement individualized screening programs are needed.

## Teaser

This cross-sectional study examines temporal shifts in age distribution of obesity-associated cancers and non–obesity-associated cancers across race/ethnicity- and sex-specific strata.

## Introduction

The International Agency for Research on Cancer reports that there is sufficient evidence that excess body adiposity is associated with increased risk for cancer in 13 anatomical sites.^1^ A study by the Centers for Disease Control and Prevention^2^ reported that 40% of all cancers diagnosed in 2014 were associated with overweight and obesity. Thus, excess body adiposity is recognized as an important modifiable risk factor to prevent multiple cancers.^3,4,5^

Overweight or obesity affects 2 billion people worldwide.^6^ It is expected that if post-2000 trends in body mass index persist, obesity prevalence will reach 18% in men and surpass 21% in women by 2025 worldwide.^7^ Using data from the National Health and Nutrition Examination Survey, a recent Centers for Disease Control and Prevention report^8^ indicated that from 1988-1994 to 2015-2016, the prevalence of obesity in the United States more than doubled in the 20- to 39-year age group (from 17.7% to 35.7%), whereas it increased from 27.9% to 42.8% in the 40- to 59-year age group and from 23.7% to 41.0% among adults 60 years or older.

Obesity that occurs during adolescence and adulthood has been implicated in cancer.^9^ Similarly, longer duration of obesity or overweight may be associated with increased risk of developing several types of cancer.^10,11^ Moreover, obesity and obesogenic diets have been shown to accelerate cancer development in murine studies, shifting its occurrence to younger ages.^12^ Therefore, obesity, especially morbid obesity, may be associated with significantly earlier onset of multiple cancers.^12,13,14,15,16,17^ Although the incidence of many of the obesity-associated cancers (OACs) has been increasing in the broader range of 20 to 74 years of age,^2^ little is known about how cancer burden is shifting across age-, race/ethnicity-, and sex-specific strata over time. Such an analysis is important to understand the epidemiologic, pathogenesis, public health, and health care implications.

## Methods

### Overview

In this cross-sectional study, to determine which specific subgroups of the population are most affected by shifts in the distribution of incident cancers, we used data from the Surveillance, Epidemiology, and End Results 18 (SEER18) database (excluding Alaska Natives) for cancer cases diagnosed from January 1, 2000, to December 31, 2016, and evaluated trends in the percentage of cases diagnosed in each of 3 age groups (20-49, 50-64, and ≥65 years) and across race/ethnicity- and sex-specific strata. We hypothesized that the greatest increase in OACs would shift to younger ages. Given the deidentified nature of the data, the Case Western Reserve University Institutional Review Board determined that this research did not involve human subjects research; thus, informed consent was not needed and the need for a waiver was not applicable. This study followed the Strengthening the Reporting of Observational Studies in Epidemiology (STROBE) reporting guideline.

We removed cases diagnosed among Alaska residents because cases in Alaska are reported only for individuals self-identified as Native Alaskans, whereas our study was limited to non-Hispanic white, non-Hispanic black, and Hispanic individuals, as noted below. For cancers listed below, including OACs and non-OACs used for comparison, we examined the distribution of incident cases by age group and in race/ethnicity- and sex-specific strata.

### Data Source

We used publicly available data from the SEER and the SEER*Stat software.^18^ SEER sites became part of the SEER program at different points in time. By 2000, data for the 17 sites in SEER18 were available for analysis. Accordingly, we conducted our analysis on data that spanned 2000 to 2016. The SEER18 covers 28% of the US population, and approximately 97% of incident cases diagnosed within the SEER regions are represented. On the basis of measures of income and education, the SEER data are representative of the US population and cover geographically diverse regions of the country. For each case, SEER registries collect patient demographics (age, sex, race, and ethnicity), month and year of diagnosis, anatomical cancer site, and detailed tumor characteristics.

### Study Population

To obtain robust estimates, especially for low-incidence cancers, and given the small number of cases in the other racial/ethnic categories, we limited our study population to individuals self-identified as non-Hispanic white, non-Hispanic black, or Hispanic who were diagnosed with incident OACs and non-OACs. We also limited our study population to persons 20 years or older, thus excluding childhood cancers and cancers occurring in adolescents. In addition, we limited breast cancer cases to women.

### Variables of Interest

Our outcome variable was age distribution of incident cases of 13 OACs and all non-OACs within race/ethnicity- and sex-specific strata over time. Data for non-OACs were used for comparison purposes. Age at diagnosis was grouped as 20 to 49, 50 to 64, and 65 years or more. Hypothesizing that age distribution might vary across race/ethnicity- and sex-specific groups, we created 6 race/ethnicity- and sex-specific strata, as follows: non-Hispanic white men, non-Hispanic black men, Hispanic men, non-Hispanic white women, non-Hispanic black women, and Hispanic women. The OACs based on the anatomical cancer site included colon and rectum (combined as colorectal), female breast, uterus, gallbladder and other biliary, esophagus, stomach, liver and intrahepatic bile duct, pancreas, ovary, kidney and renal pelvis, thyroid, and myeloma. Cancers not identified as OACs were grouped as non-OACs. Except when referring to specific OAC(s) and non-OAC(s), all results reflect all OACs or non-OACs combined.

### Statistical Analysis

**Figure 1.**
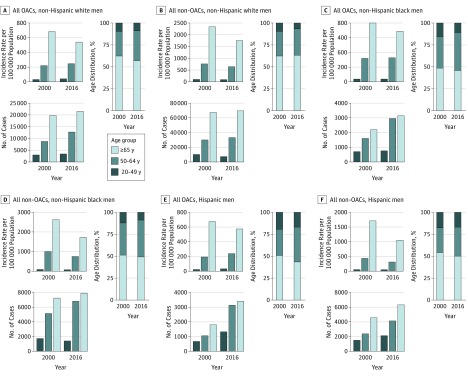
Comparison of Obesity-Associated Cancers (OACs) and Non-OACs in Men by Age- and Race/Ethnicity-Specific Strata Incidence rate per 100 000 persons, total number of incident cases, and case distribution across age groups are shown for all men.

**Figure 2.**
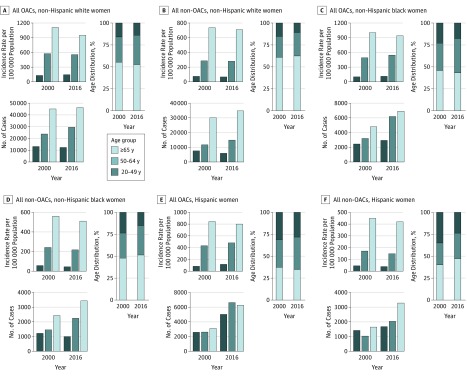
Comparison of Obesity-Associated Cancers (OACs) and Non-OACs in Women by Age- and Race/Ethnicity-Specific Strata Incidence rate per 100 000 persons, total number of incident cases, and case distribution across age groups are shown for all women.

Data were analyzed from August 1, 2018, to June 30, 2019. We used SEER*Stat software, version 8.3.5 (National Cancer Institute) to create data tables that contain the variables of interest. We then exported these tables into SAS software, version 9.4 (SAS Institute Inc) for analysis. For each of the cancers of interest and race/ethnicity- and sex-specific stratum, we calculated the distribution of incident cases across the 3 age groups (20-49, 50-64, and ≥65 years). We also obtained incidence rates for these cancers from SEER*Stat, which uses population estimates from the US Census. Figure 1 and Figure 2 show the number of incident cases, incidence rates, and distribution by age group in 2000 and 2016 for OACs and non-OACs by age-, race/ethnicity-, and sex-specific strata.

To test the statistical significance of the changes in age distribution observed through data visualization, we developed race/ethnicity- and sex-stratified logistic regression models and examined the interaction term of OAC by year for each age group. The analytic data set for these regression models included, for each race/ethnicity- and sex-specific stratum, the variables year (coded as year 2000, treated as a continuous variable), OAC (0 or 1), and the number of cases by age group. Three sets of regression analysis were conducted, with 1 for each age group. In separate models, we defined our outcome variable or event as 1 for the 20 to 49 years, 50 to 64 years, and 65 years or older age groups and 0 for the other 2 age groups combined. For example, when analyzing an event for the age group 20 to 49 years, the outcome variable *y* was coded as 1 for the age group of 20 to 49 years and the corresponding number of incident cases and 0 for the other 2 age groups combined and the corresponding number of cases. The independent variables were year, OAC, and the interaction term year by OAC. A positive coefficient for the interaction term indicated that the slope for OAC was higher than that for non-OAC. In the eFigure in the Supplement, we present the percentage changes in the absolute number of cases for select OACs and non-OACs from 2000 to 2016.

## Results

The study population included 2 665 574 incident OAC cases (70.3% women) and 3 448 126 incident non-OAC cases (32.0% women) diagnosed from 2000 to 2016 among SEER site residents. Among OAC cases, the high incidence among women was attributable to breast, ovarian, and uterine cancer cases.

In Figure 1 and Figure 2, we present age-specific data for OACs and non-OACs diagnosed in 2000 and 2016, stratified by race/ethnicity and sex. We also present the incidence rates and the absolute number of cases. In most cases, we observed a decrease in the incidence rates but an increase in the number of incident cases among individuals 65 years or older as a function of the aging population. In non-Hispanic white men, for example, the incidence rate for OACs decreased from 684.5 per 100 000 persons in 2000 to 541.7 per 100 000 persons in 2016, but the number of cases increased from 19 743 to 21 448. For the younger age group (20-49 years), the incidence rates (ie, cases per 100 000 population) increased or remained relatively stable, but the absolute number of incident cases increased. In Hispanic women, for example, the incidence rate for OACs increased from 82.3 to 115.0, but the number of cases increased from 2599 to 5058 (Figure 2E). In particular, and especially for OACs, shifts in the age distribution occurred, with a decrease in the percentage of incident cases in the 65 years or older group and an increase in the percentage of cases in the 50- to 64-year age group. In Hispanic men with OACs, for example, there was a decrease in the percentage of individuals 65 years or older from 50.7% to 43.3% and an increase in the percentage of individuals 50 to 64 years old from 29.8% to 39.8% (Figure 1E). For OACs, we observed increases in the percentage of cases in individuals 50 to 64 years of age among non-Hispanic black men (from 35.8% to 43.1%), Hispanic men (from 29.8% to 39.8%), and non-Hispanic black women (from 30.9% to 38.8%).

With regard to non-OACs, although the patterns in incidence rates, number of incident cases, and age distribution were similar to those in OACs, the shift in age distribution in non-OACs was less pronounced, suggesting that the increase in the percentage of incident cases among middle-aged adults was greater in OACs than in non-OACs. Among non-Hispanic white men, for example, the percentage of cases in the 50- to 64-year age group increased from 28.0% to 30.2% for non-OAC cases and from 27.8% to 34.0% for OAC cases.

The Table gives the odds ratios (ORs) for the annual change in the percentage of cases belonging to a given age group for OACs and non-OACs across race/ethnicity- and sex-specific strata that were assessed using race/ethnicity- and sex-specific stratified logistic regression analysis, as well as the ratio of OAC to non-OAC ORs, corresponding to the OR obtained from the year by OAC interaction term in the logistic model. For both OACs and non-OACs, the percentage of cases in the 20- to 49-year age group decreased significantly over time in all race/ethnicity- and sex-specific strata. For example, for non-Hispanic white men, the annual change in odds for the 20- to 49-year age group decreased by 0.6% for OACs and decreased by 2.5% for non-OACs. The ratio of OAC to non-OAC ORs of 1.020 (95% CI, 1.018-1.022) implies that non-OACs decreased to a greater extent than OACs. The ratio of OAC to non-OAC ORs was greater than 1.000 for all except Hispanic men, for whom it was lower than 1.000 (0.993; 95% CI, 0.989-0.998). For non-Hispanic black men, the ratio of OAC to non-OAC ORs was 1.000 (95% CI, 0.996-1.005).

**Table.** Odds Ratios (95% CIs) for the Annual Change in the Proportions of OAC and Non-OAC Cases in a Given Age Group

Group	OR (95% CI)		OAC to Non-OAC Ratio of ORs (95% CI)^a^
	OAC	Non-OAC	
**Men**			
Age 20-49 y			
Non-Hispanic white	0.994 (0.993-0.996)	0.975 (0.974-0.976)	1.020 (1.018-1.022)
Non-Hispanic black	0.978 (0.974-0.982)	0.978 (0.975-0.980)	1.000 (0.996-1.005)
Hispanic	0.988 (0.985-0.992)	0.995 (0.992-0.997)	0.993 (0.989-0.998)
Age 50-64 y			
Non-Hispanic white	1.019 (1.018-1.021)	1.006 (1.006-1.007)	1.013 (1.012-1.014)
Non-Hispanic black	1.023 (1.020-1.025)	1.018 (1.016-1.019)	1.005 (1.002-1.008)
Hispanic	1.026 (1.023-1.028)	1.015 (1.013-1.017)	1.010 (1.007-1.014)
Age ≥65 y			
Non-Hispanic white	0.985 (0.984-0.986)	1.002 (1.002-1.003)	0.983 (0.981-0.984)
Non-Hispanic black	0.988 (0.986-0.991)	0.992 (0.990-0.994)	0.996 (0.993-0.999)
Hispanic	0.984 (0.981-0.986)	0.990 (0.988-0.992)	0.994 (0.990-0.997)
**Women**			
Age 20-49 y			
Non-Hispanic white	0.988 (0.987-0.989)	0.972 (0.971-0.974)	1.016 (1.015-1.018)
Non-Hispanic black	0.978 (0.976-0.980)	0.964 (0.961-0.967)	1.015 (1.011-1.019)
Hispanic	0.990 (0.988-0.991)	0.971 (0.968-0.974)	1.019 (1.015-1.022)
Age 50-64 y			
Non-Hispanic white	1.015 (1.014-1.016)	1.011 (1.010-1.012)	1.004 (1.003-1.005)
Non-Hispanic black	1.023 (1.022-1.025)	1.018 (1.015-1.021)	1.005 (1.002-1.009)
Hispanic	1.017 (1.015-1.019)	1.015 (1.012-1.018)	1.002 (0.999-1.006)
Age ≥65 y			
Non-Hispanic white	0.994 (0.993-0.994)	1.005 (1.004-1.006)	0.989 (0.988-0.990)
Non-Hispanic black	0.993 (0.992-0.995)	1.007 (1.005-1.010)	0.986 (0.983-0.989)
Hispanic	0.993 (0.991-0.995)	1.012 (1.009-1.015)	0.981 (0.978-0.984)

Abbreviations: OAC, obesity-associated cancer; OR, odds ratio.

^a^ A ratio greater than 1.000 means that the annual change is increasing to a greater extent (or decreasing to a lesser extent) for OACs than for non-OACs. A ratio less than 1.000 means that the annual change is decreasing to a greater extent (or increasing to a lesser extent) for OACs than for non-OACs. Because of rounding, the upper or lower limit in the 95% CI may be the same as the point estimate. A ratio of 1.000 means that the annual change is increasing or decreasing for OACs and for non-OACs to the same extent.

For OACs and non-OACs, we observed a statistically significant increase over time in the percentage of cases in the 50- to 64-year age group. This finding was consistent across all race/ethnicity- and sex-specific strata. The ratio of OAC to non-OAC ORs was greater than 1.000, indicating that the increase was greater for OACs than for non-OACs. The highest increase was observed for OACs in Hispanic men, at 2.6% per year (OR, 1.026; 95% CI, 1.023-1.028), compared with an observed 1.5% per year increase in non-OAC cases. The ratio of OAC to non-OAC ORs for Hispanic men was 1.010 (95% CI, 1.007-1.014), indicating a 1% higher increase in OACs than in non-OACs. The only group in which the ratio of OAC to non-OAC ORs was not statistically significant was Hispanic women (OR, 1.002; 95% CI, 0.999-1.006).

In the age group older than 65 years, we observed a decrease over time (ORs <1.000) in OACs for both men and women across all race/ethnicity-specific strata, whereas for non-OACs, the percentage of cases increased over time in non-Hispanic white men and in women across all race/ethnicity-specific strata. The ratio of OAC to non-OAC ORs was consistently less than 1.000 for both men and women, indicating that whereas the OAC group experienced a decrease in this age group, the non-OACs experienced either a smaller decrease or an increase over time. For example, in non-Hispanic black men, the annual change in the ORs associated with that age group decreased annually by 1.2% for OACs and by 0.8% for non-OACs, and the ratio of OAC to non-OAC ORs was 0.996 (95% CI, 0.993-0.999), implying a 0.4% greater annual decrease for OACs than for non-OACs. Together, these data show a shift of OACs from the age group of 65 years or older to the younger age groups.

To assess the association of these shifts with the absolute number of incident cancer cases in the population, we evaluated the percentage change in the number of incident OACs and select non-OACs between 2000 and 2016 (eFigure in the Supplement). Across all age groups and across all race/ethnicity- and sex-specific strata, the percentage increase in the absolute number of OAC cases ranged from 7.7% in non-Hispanic white women to 123.4% in Hispanic men. For non-OACs, the range was 1.9% in non-Hispanic white men to 70.1% in Hispanic women, indicating a greater increase for OACs than for non-OACs. The greatest increase in the absolute number of OACs occurred among individuals 50 to 64 years of age, ranging from 25.3% in non-Hispanic white women to 197.8% in Hispanic men. In contrast, for non-OACs, the percentage change in the absolute number of cases in the same age group ranged from 9.7% in non-Hispanic white men to 96.7% in Hispanic women.

In non-Hispanic white men, overall, the greatest increase was for liver, thyroid, and gallbladder cancers in 50- to 64-year-old individuals (200.6% for liver, 157.2% for thyroid, and 89.7% for gallbladder). Among men 65 years or older, the greatest increase was observed for thyroid cancer (168.4%) and melanoma (128.2%). In non-Hispanic white men younger than 50 years, we observed increases for thyroid (62.1%), kidney (48.1%), and colorectal (4.8%) cancers.

For non-Hispanic black men, the number of cases more than doubled in persons 50 to 64 years old and 65 years or older for multiple cancers, including gallbladder (by 288.9% in those 50-64 years of age and 207.4% in those ≥65 years of age), liver (by 227.3% in those 50-64 years of age and 229.0% in those ≥65 years of age), thyroid (by 172.4% in those 50-64 years of age and 206.7% in those ≥65 years of age), and kidney cancers (by 125.3% in those 50-60 years of age and 113.1% in those ≥65 years age). The number of cases of myeloma increased by 98% or more but only in the older age groups. Among those 20 to 49 years of age, we observed increases for thyroid and kidney cancers and for myeloma, which had the greatest increase in this age group.

Among non-Hispanic white women, the greatest increase in the number of cases occurred in the 50- to 64-year age group (thyroid, liver, melanoma, and kidney cancers). Similar increases for thyroid cancer and melanoma were observed in the oldest age group. In non-Hispanic black women, increases by 2- or more than 3-fold were observed for several cancers, including liver, thyroid, and uterine cancers (for both the 50- to 64-year and ≥65-year age groups). Similarly, the number of cases more than doubled for gallbladder, pancreas, and stomach cancers.

In Hispanic men, the number of cases of thyroid cancer increased by 179.5% in the 20- to 49-year age group but by more than 300% in the older age groups. Similarly, the number of liver cancer cases increased by more than 350% in the 50- to 64-year age group but by less than 200% in the oldest age group. The number of cases of kidney cancer increased by more than 200% across all age groups. We observed similar increases for several other cancers, including oral cavity and pharynx, colorectal, brain, and stomach. Overall, however, we observed the greatest increase for all OACs combined in the 50- to 64-year age group (197.8% vs 93.9% in the youngest age group and 91.0% in the oldest age group).

In Hispanic women, we observed an approximately 400% increase in the number of thyroid cancer cases in the 50- to 64-year age group; a more than 200% increase in the number of melanomas and kidney, uterine, and pancreatic cancers; and a 150% to 200% increase for liver and brain cancers and myeloma. The number of incident cases of several cancers also increased in the 20- to 49-year age group, including kidney, thyroid, pancreatic, and uterine cancers and myeloma. In the age group of 65 years or older, the greatest increases were observed for thyroid cancer and melanoma.

## Discussion

In this study, we hypothesized that because obesity is associated with the development of cancer,^12^ a shift of incident OACs might occur from older to younger individuals and that this shift might be contributing to the increase in the number of people younger than 50 with cancer.^12^ Although we observed a shift in the distribution of age to 50 to 64 years, there was an increase in the absolute number of incident cancers, many of which were OACs, in persons 20 to 49 years of age, especially among non-Hispanic black and Hispanic men and women. Our finding that the percentage of OACs diagnosed among those 65 years or older is decreasing contrasts with previous projections of a marked increase cancer burden in older people.^19,20,21,22^

The shift of cancer burden to younger age groups has important public health, research, and policy implications. First, earlier onset of cancer may be associated with more advanced-stage breast or colorectal cancer at diagnosis,^23,24^ as well as more aggressive cancer,^25,26^ premature mortality, and more years of potential life lost. It is possible that changes in cancer surveillance over time have improved early cancer detection. Nonetheless, these trends have prompted concerns about risk stratification to tailor the timing of screening based on the individual’s personal, family, and genetic risk factors to initiate early screening.^24^ For example, in the case of colorectal cancer, uptake of screening and other interventions for risk factor modification (eg, smoking and red meat consumption) have been associated with decreases in the overall incidence rates in past decades, especially in those 65 years or older.^27,28^ In contrast, incidence rates increased in the younger than 50 years group during the same period^27^—a trend that may reflect the lack of a personalized approach to cancer screening in younger individuals. Second, although a prior study^29^ identified older adults (ie, ≥75 years old) as the demographic group that constitutes most cancer survivors until 2040, our findings suggest that greater cancer burden among individuals younger than 65 years means that more incoming and younger Medicare beneficiaries will be cancer survivors with a high burden of comorbidity, mental health problems, functional impairment,^30,31^ financial hardship, and medical bankruptcy.^32,33^ Because of these hardships, many may also qualify for Medicaid. Together, these findings suggest an increasing cancer burden on Medicare and Medicaid programs in the future.

Our finding that the burden of cancer is shifting to younger age groups is consistent with previous studies.^2,34^ However, although prior studies^34,35^ have reported on the increase in incidence rates among younger adults, our study examined changes in the distribution of incident cancer cases across multiple age groups over time and found that the increase in incidence in younger adults was accompanied by a concurrent decrease in older adults. This finding is despite the fact that, in our study, the size of the population increased for those 50 to 64 years of age (by 51.8%) and those 65 years or older (by 42.8%), whereas the 20- to 49-year-old population increased by only 3.6%. Different analyses yield different insights, and this novel approach to quantify the shift of OACs in age and across different race/ethnicity groups yields findings that may be more useful to inform public health strategies. We believe that although our analysis of the changes in incidence rates in young adults provide a longer-term outlook on trends in cancer burden, our analysis by age distribution helps us to identify individuals in certain age groups who are currently experiencing a disproportionate burden of cancer and to more appropriately target cancer and prevention control efforts in the immediate future.

While the present article was in preparation, a study^36^ reported that the incidence of OACs has been increasing in younger birth cohorts, ranging from 1.6 times for multiple myeloma to 4.9 times for kidney cancer. Findings from that study^36^ support our work. This recent report^36^ plus our current study indicate the importance of this problem.

### Strengths and Limitations

Our study has a number of strengths, the most important of which is the inclusion of data spanning a period of 17 years, which yielded a large number of cases and robust estimates for most of the cancers studied. Nonetheless, some of the cancers (eg, liver and gallbladder cancers) had small numbers, with fluctuations from year to year. Limitations include the lack of data on body adiposity (eg, body mass index) in SEER. Thus, we were unable to determine whether OACs actually occurred in individuals with high body adiposity. Similarly, we were unable to account for factors such as poverty, racism, compromised social determinants of health, smoking and other behaviors, environmental factors, genetic mutations, and family history of cancer, all of which are associated with increased cancer risk. The availability of data on these risk factors in cancer surveillance databases will augment the utility of such data resources.

## Conclusions

Our trend analysis spanning the 17-year period of 2000 to 2016 indicated a shift in the number of OACs to younger age groups. The findings suggest that public health interventions are needed to prevent and reduce obesity and other known risk factors, to implement individualized screening strategies, and to disrupt the obesity-cancer association.

## Notes

Supplement. **eFigure.** Percent Change in the Number of Cases Between Years 2000 and 2016 by Age and Race/Ethnicity- and Sex-Strata
