# Supplementary material for: Changes in Age Distribution of Obesity-Associated Cancers
Source: JAMA Netw Open. 2019 Aug 14;2(8):e199261. doi: 10.1001/jamanetworkopen.2019.9261 (PMC6694395; doi:10.1001/jamanetworkopen.2019.9261)
Supplement: Supplement. — eFigure. Percent Change in the Number of Cases Between Years 2000 and 2016 by Age and Race/Ethnicity- and Sex-Strata [file jamanetwopen-2-e199261-s001.pdf]

Supplementary Online Content

Koroukian SM, Dong W, Berger NA. Changes in age distribution of obesity-associated cancers. *JAMA Netw Open*. 2019;2(8):e199261. doi:10.1001/jamanetworkopen.2019.9261

eFigure. Percent Change in the Number of Cases Between Years 2000 and 2016 by Age and Race/Ethnicity- and Sex-Strata

This supplementary material has been provided by the authors to give readers additional information about their work.

© 2019 Koroukian SM et al. *JAMA Network Open*.

Non-Hispanic White Male

Cancer Site	20-49 yrs	50-64 yrs	65+ yrs	All Age Groups
Thyroid	62.1	157.2	168.4	118.9
Liver & IBD	-42.7	200.6	93.2	110.3
Melanoma of the Skin	-20.4	74.9	128.2	73.9
Kidney & Renal Pelvis	48.1	70.2	62.9	63.6
Gallbladder & OB	-15.4	89.7	49.7	53.0
Pancreas	-17.4	56.6	56.4	51.3
Oral Cavity & Pharynx	-24.2	67.4	61.4	49.0
Myeloma	-4.5	57.3	48.9	47.5
CLL	-13.1	39.0	35.1	33.3
Esophagus	-38.8	30.8	34.6	27.2
Urinary Bladder	-42.6	3.9	19.4	12.4
Brain & ONS	-19.5	27.2	21.2	10.6
Testis	-5.9	63.7	48.1	0.9
Stomach	-31.8	7.7	-4.1	-3.0
Lung & Bronchus	-59.9	-13.7	-7.8	-11.8
Colon & Rectum	4.8	9.9	-30.9	-17.7
All OACs	11.1	45.3	8.6	19.1
All NonOACs	-28.1	9.7	3.0	1.9

Non-Hispanic White Female

Cancer Site	20-49 yrs	50-64 yrs	65+ yrs	All Age Groups
Thyroid	55.1	167.3	150.9	100.0
Liver & IBD	0.0	202.9	48.5	71.5
Melanoma of the Skin	-1.0	104.0	92.2	57.1
Kidney & Renal Pelvis	39.3	86.9	31.3	46.8
Uterine	-10.8	53.1	28.0	32.3
Pancreas	0.0	66.0	24.7	30.8
Myeloma	26.7	57.4	15.3	24.6
Oral Cavity & Pharynx	-13.5	54.2	15.0	21.8
Gallbladder & OB	-3.7	46.1	16.9	21.2
CLL	0.0	47.7	15.0	20.7
Brain & ONS	-25.6	26.7	13.8	6.5
Lung & Bronchus	-49.3	1.9	11.7	6.0
Esophagus	-51.4	30.5	2.8	5.4
Breast	-20.9	11.8	11.3	5.2
Urinary Bladder	-44.0	4.6	4.2	1.4
Stomach	2.0	53.8	-16.4	-4.4
Cervix Uteri	-29.7	9.6	-20.4	-18.3
Ovary	-32.7	-17.1	-18.8	-20.6
Colon & Rectum	23.5	11.1	-35.2	-22.9
All OACs	-5.9	25.3	2.5	7.7
All NonOACs	-21.8	27.6	15.2	12.4

Non-Hispanic Black Male

Cancer Site	20-49 yrs	50-64 yrs	65+ yrs	All Age Groups
Gallbladder & OB		288.9	207.4	228.6
Liver & IBD	-56.1	227.3	229.0	167.9
Thyroid	27.5	172.4	206.7	109.5
Kidney & Renal Pelvis	66.4	125.3	113.1	108.0
Myeloma	45.5	98.4	126.2	105.2
Pancreas	-29.5	84.5	56.3	54.8
CLL	8.3	52.4	58.3	51.6
Urinary Bladder	-13.6	53.6	55.1	48.7
Testis	20.4			45.6
Colon & Rectum	11.1	57.8	-0.9	20.1
Brain & ONS	-6.3	39.5	21.4	14.9
Oral Cavity & Pharynx	-32.4	11.2	42.1	9.5
Stomach	-30.0	18.0	12.5	8.0
Lung & Bronchus	-65.8	1.4	15.2	2.4
Melanoma of the Skin			57.1	-7.3
Esophagus	-61.3	-17.8	1.4	-13.3
All OACs	7.2	81.7	43.0	51.2
All NonOACs	-17.3	30.7	9.0	13.8

Non-Hispanic Black Female

Cancer Site	20-49 yrs	50-64 yrs	65+ yrs	All Age Groups
Liver & IBD	-5.0	316.7	182.0	191.5
Thyroid	111.7	271.4	172.2	166.3
Uterine	67.6	198.3	120.9	139.4
Kidney & Renal Pelvis	68.3	160.2	117.2	122.9
Gallbladder & OB		188.0	70.8	98.0
Myeloma	22.9	119.0	89.7	89.0
Pancreas	66.7	106.9	35.8	54.7
CLL		136.8	33.3	48.3
Breast	0.0	78.4	63.9	47.8
Urinary Bladder	-22.7	94.0	39.8	44.8
Oral Cavity & Pharynx	-18.2	104.6	33.3	39.2
Ovary	25.0	45.7	26.5	32.3
Lung & Bronchus	-52.3	33.6	46.4	31.0
Melanoma of the Skin			40.0	22.9
Brain & ONS	4.4	39.4	20.8	19.8
Stomach	29.7	103.0	-17.9	10.3
Colon & Rectum	37.9	44.8	-13.6	8.9
Cervix Uteri	-29.3	17.5	-30.8	-18.7
Esophagus		0.0	-30.9	-19.0
All OACs	18.1	92.1	44.2	52.9
All NonOACs	-16.6	53.3	40.1	30.4

Hispanic Male

Cancer Site	20-49 yrs	50-64 yrs	65+ yrs	All Age Groups
Thyroid	179.5	300.0	330.4	233.1
Kidney & Renal Pelvis	214.2	225.6	205.8	215.0
Liver & IBD	-23.9	355.9	173.8	191.6
Pancreas	112.5	244.7	95.7	135.9
Gallbladder & OB	40.0	216.7	125.4	134.7
Melanoma of the Skin	66.7	74.0	206.8	113.7
Oral Cavity & Pharynx	24.7	157.9	115.4	105.8
Myeloma	21.4	135.8	109.0	100.0
Testis	92.3	100.0		91.7
Colon & Rectum	87.1	167.3	42.0	85.6
Brain & ONS	27.7	153.5	125.0	80.2
Esophagus	5.3	58.8	109.1	78.2
Urinary Bladder	14.6	62.8	93.0	77.5
Stomach	58.6	103.5	34.3	56.7
Lung & Bronchus	-30.4	43.9	45.8	39.2
CLL		55.2	32.1	34.7
All OACs	93.9	197.8	91.0	123.4
All NonOACs	42.0	73.6	38.2	48.9

Hispanic Female

Cancer Site	20-49 yrs	50-64 yrs	65+ yrs	All Age Groups
Thyroid	160.0	379.6	275.8	214.2
Kidney & Renal Pelvis	221.4	243.2	159.7	201.4
Uterine	129.6	232.3	151.7	176.6
Liver & IBD	55.6	176.4	150.4	148.9
Pancreas	200.0	202.7	115.6	143.6
Myeloma	187.5	183.7	107.8	140.0
Esophagus			140.9	125.8
CLL		45.5	148.1	102.3
Melanoma of the Skin	6.7	224.4	217.1	100.5
Breast	60.4	116.1	122.3	97.4
Lung & Bronchus	26.6	94.2	106.9	95.5
Oral Cavity & Pharynx	41.0	144.2	90.3	93.1
Ovary	50.0	109.4	91.5	82.4
Colon & Rectum	162.9	150.2	31.3	81.9
Stomach	110.8	132.6	45.5	81.6
Gallbladder & OB	-8.3	108.3	88.3	80.6
Brain & ONS	9.6	193.9	63.5	62.5
Urinary Bladder	12.5	39.6	72.2	56.1
Cervix Uteri	-7.8	11.6	12.1	-0.7
All OACs	94.6	153.0	102.0	115.8
All NonOACs	17.4	96.7	99.0	70.1

High

Low

Not shown due to small number of cases (<11)

Liver & IBD: Liver and Intrahepatic Bile Duct

Gallbladder & OB: Gallbladder and Other Biliary

Brain & ONS: Brain and Other Nervous System

CLL: Chronic Lymphocytic Leukemia

© 2019 Koroukian SM et al. JAMA Network Open.
